# A nearby long gamma-ray burst from a merger of compact objects

**DOI:** 10.1038/s41586-022-05327-3

**Published:** 2022-12-07

**Authors:** E. Troja, C. L. Fryer, B. O’Connor, G. Ryan, S. Dichiara, A. Kumar, N. Ito, R. Gupta, R. T. Wollaeger, J. P. Norris, N. Kawai, N. R. Butler, A. Aryan, K. Misra, R. Hosokawa, K. L. Murata, M. Niwano, S. B. Pandey, A. Kutyrev, H. J. van Eerten, E. A. Chase, Y.-D. Hu, M. D. Caballero-Garcia, A. J. Castro-Tirado

**Affiliations:** 1 Department of Physics, University of Rome “Tor Vergata”, Rome, Italy; 2 School of Earth and Space Exploration, Arizona State University, Tempe, AZ USA; 3 Center for Theoretical Astrophysics, Los Alamos National Laboratory, Los Alamos, NM USA; 4 Department of Physics, The George Washington University, Washington, DC USA; 5 Astronomy, Physics and Statistics Institute of Sciences (APSIS), The George Washington University, Washington, DC USA; 6 Department of Astronomy, University of Maryland, College Park, MD USA; 7 Astrophysics Science Division, NASA Goddard Space Flight Center, Greenbelt, MD USA; 8 Perimeter Institute for Theoretical Physics, Waterloo, Ontario Canada; 9 Department of Astronomy and Astrophysics, The Pennsylvania State University, University Park, PA USA; 10 Aryabhatta Research Institute of Observational Sciences (ARIES), Nainital, India; 11 School of Studies in Physics and Astrophysics, Pandit Ravishankar Shukla University, Chattisgarh, India; 12 Department of Physics, University of Warwick, Coventry, UK; 13 Department of Physics, Tokyo Institute of Technology, Tokyo, Japan; 14 Department of Physics, Deen Dayal Upadhyaya Gorakhpur University, Gorakhpur, India; 15 Department of Physics, Boise State University, Boise, ID USA; 16 Physics Department, University of Bath, Bath, UK; 17 Instituto de Astrofísica de Andalucía (IAA), CSIC, Granada, Spain; 18 Unidad Asociada al CSIC Departamento de Ingeniería de Sistemas y Automática, Escuela de Ingeniería Industrial, Universidad de Málaga, Málaga, Spain

**Keywords:** High-energy astrophysics, Compact astrophysical objects

## Abstract

Gamma-ray bursts (GRBs) are flashes of high-energy radiation arising from energetic cosmic explosions. Bursts of long (greater than two seconds) duration are produced by the core-collapse of massive stars^1^, and those of short (less than two seconds) duration by the merger of compact objects, such as two neutron stars^2^. A third class of events with hybrid high-energy properties was identified^3^, but never conclusively linked to a stellar progenitor. The lack of bright supernovae rules out typical core-collapse explosions^4–6^, but their distance scales prevent sensitive searches for direct signatures of a progenitor system. Only tentative evidence for a kilonova has been presented^7,8^. Here we report observations of the exceptionally bright GRB 211211A, which classify it as a hybrid event and constrain its distance scale to only 346 megaparsecs. Our measurements indicate that its lower-energy (from ultraviolet to near-infrared) counterpart is powered by a luminous (approximately 10^42^ erg per second) kilonova possibly formed in the ejecta of a compact object merger.

## Abstract (web summary)

A bright, long-duration gamma-ray burst observed by the Swift observatory has hybrid high-energy properties, suggesting that its origin is the merger of a compact binary.

## Main

**Fig. 1:**
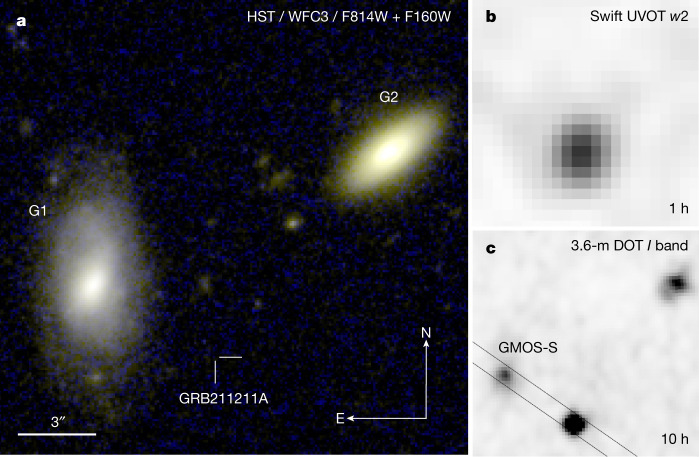
The field of GRB 211211A. **a**, False colour image combining optical (F814W; blue) and near-infrared (F160W; red and green) HST observations of GRB 211211A, carried out with the Wide Field Camera 3 (WFC3) camera in April 2022 (approximately 4 months after the burst). Two bright galaxies (G1 at *z* ≈ 0.0762, and G2 at *z* ≈ 0.4587) and several fainter ones are visible, but no source is detected at the location of GRB 211211A. The most probable host galaxy is G1, a low-mass, late-type galaxy. The projected physical offset between the burst and the centre of the galaxy is approximately 8 kpc, one of the largest ever measured for a long burst. **b**,**c**, The same field is shown in the UV *w*2 filter observed by Swift at 1 h after the burst (**b**), and in the optical *I* filter acquired by the 3.6-m DOT/4K × 4K CCD imager at 10 h after the burst (**c**). The solid lines show the slit position used for optical spectroscopy with Gemini/GMOS-S. The bright UV counterpart rules out a high-redshift origin, whereas its rapid reddening is consistent with the onset of a kilonova.

**Extended Data Fig. 1:**
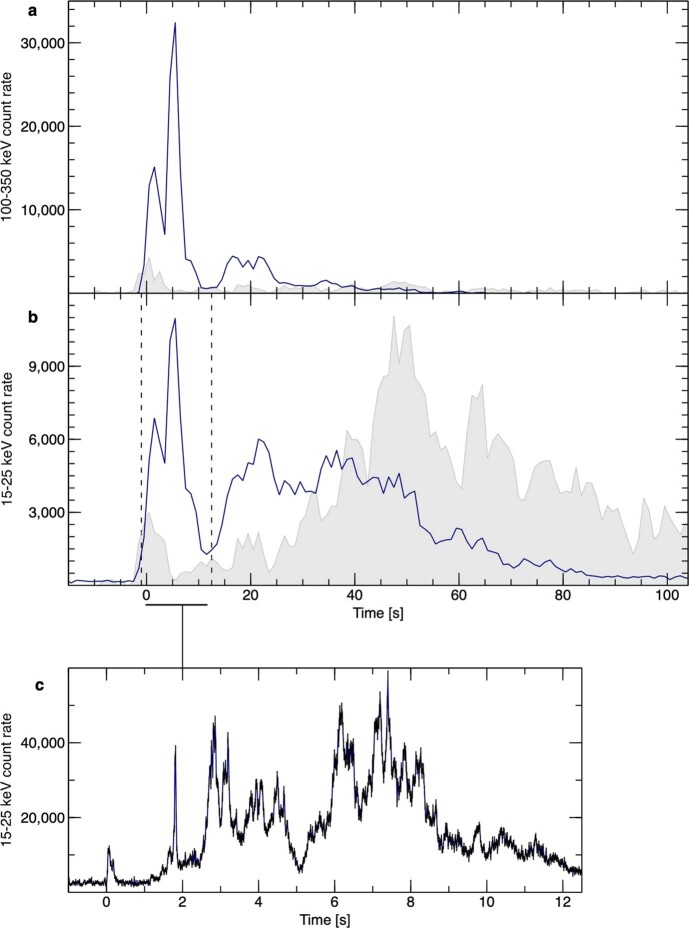
Prompt gamma-ray phase of GRB 211211A. **a**,**b**, The Swift background subtracted light curves of GRB 211211A are shown in two energy bands and compared with the time history of GRB 060614 (grey shaded area) rescaled at a distance of 346 Mpc. The time bin is 1 s. Error bars are 1*σ*. Both bursts display a first episode with hard spectrum (dominant in GRB 211211A), followed by a long-lasting tail with soft spectrum (dominant in GRB 060614). **c**, The inset magnifies the first 12 s, showing a weak precursor at *T*_0_ preceding the main prompt event. The time bin is 16 ms.

On 11 December 2021 at 13:59:09 Universal Time (ut; hereafter *T*_0_), NASA’s Neil Gehrels Swift observatory (hereafter Swift) discovered GRB 211211A^9^ as an extremely bright burst with a duration of over 50 s (Extended Data Fig. 1). The burst was independently observed by the Fermi, INTEGRAL and CALET satellites. Its optical, ultraviolet (UV) and X-ray counterparts were localized within minutes, close to a nearby galaxy, SDSS J140910.47+275320.8 (G1 in Fig. 1), at a distance of 346 Mpc (Methods). Spectroscopic observations of the optical counterpart showed a featureless continuum^10^ and did not allow for a direct measurement of the GRB distance scale. However, when combined with the detection of a bright UV counterpart, these observations point to a low-redshift origin for GRB 211211A (*z* < 1.5 at the 99.9% confidence level, CL; Methods).

Despite the close distance of the GRB, deep imaging with the Hubble Space Telescope (HST; Fig. 1) does not detect any underlying host galaxy down to F160W > 27.6 AB mag. Several extended objects are visible within 10 arcsec from the GRB position; however, their probability of chance superposition is high (greater than 10%; see Methods). The most probable birthsite is in the outskirts of the nearby galaxy G1, at a projected physical offset of 8.00 ± 0.04 kpc from the nucleus of the galaxy. This association is also supported a) by probabilistic arguments; the chance alignment between the GRB and the bright G1 galaxy is only 1.4%, b) by the uncommon brightness of the prompt gamma-ray emission; the total fluence is approximately 3 × 10^−4^ erg  cm^−2^ (15–150 keV), the second-highest value recorded by Swift^11^, and c) by the faintness of the X-ray counterpart, as the X-ray flux to gamma-ray fluence ratio at 11 hr, log *f*_X,11hr_/*F*_γ_ ≈ −7.9, lies below the typical GRB distribution^12^ as expected for an explosion in a rarefied circumburst medium^13,14^ (Methods).

**Extended Data Fig. 2:**
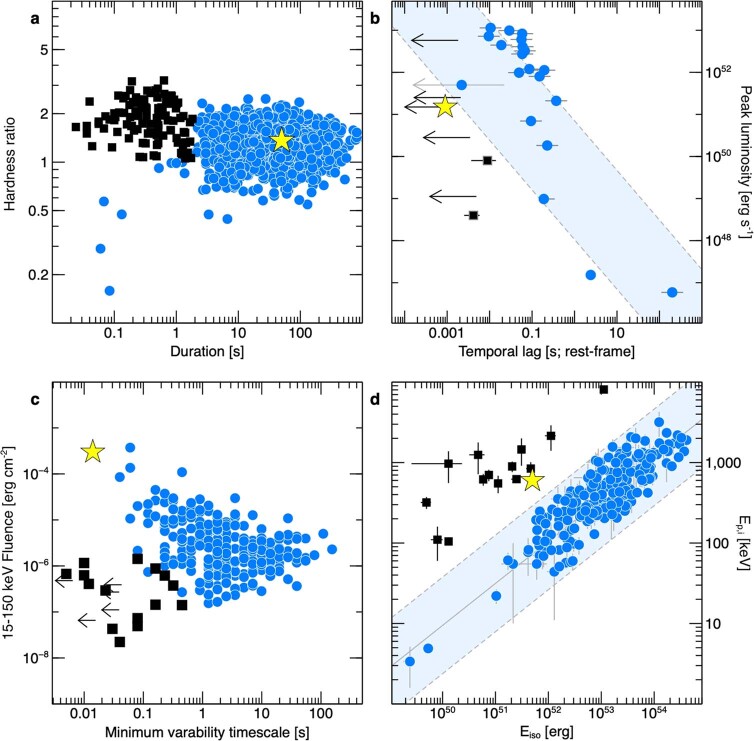
GRB classification scheme. **a**–**d**, The traditional GRB classification, based on the duration/hardness ratio diagram (**a**), is not unambiguous. Additional classifiers, used to break the degeneracy, are the lag–luminosity relation (**b**), the variability timescale (**c**) and the Amati relation (**d**). Long GRBs (circles) and short GRBs (squares) occupy different regions of these plots. Dashed lines show the boundaries of the long GRB regions (shaded areas). GRB 211211A (star symbol) belongs to the class of long soft bursts (**a**), but its other high-energy properties are common among short GRBs. Error bars represent 1*σ*; upper limits (arrows) are 3*σ*.

**Extended Data Fig. 4:**
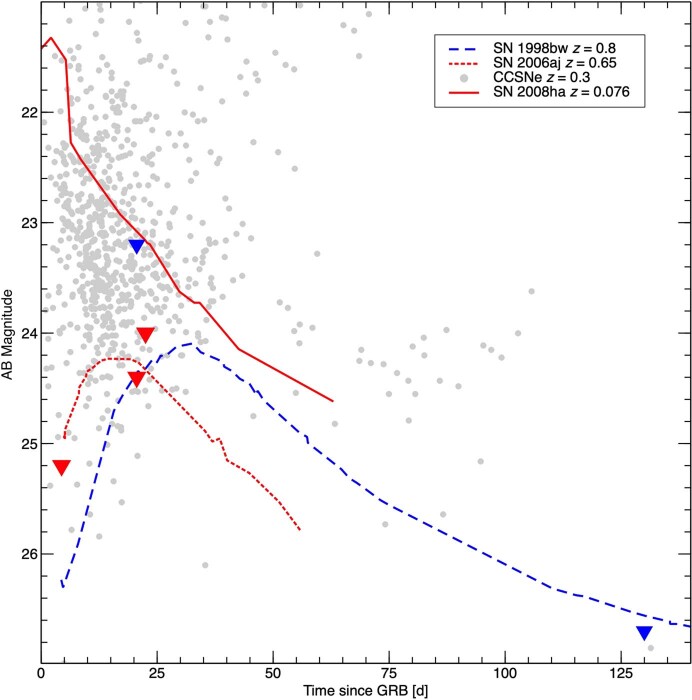
No supernova associated with GRB 211211A. Optical upper limits (representing 3*σ* CL) in the r-band (red) and i-band (blue) rule out the presence of any known supernova following GRB 211211A. Bright SNe associated with GRBs, such as SN 1998bw^15^ and SN 2006aj^75^, would have been detected up to *z* = 0.8 (dashed line) and *z* = 0.65 (dotted line), respectively. Symbols show the peak magnitude of core-collapse supernovae from the ZTF Bright Transient Survey Sample^76^ rescaled at *z* = 0.3, demonstrating that most ordinary SNe were detectable up to this distance. At the distance *z* ≈ 0.076 of the putative host galaxy, the faint SN 2008ha^6^ (solid line) is also ruled out.

The association with a galaxy at 346 Mpc implies that GRB 211211A is one of the closest long bursts ever discovered, yet the properties of its gamma-ray emission—such as the negligible temporal lag, short variability timescale and hard spectrum—do not fit into this class of events (Extended Data Fig. 2). These are distinctive features of short bursts and classify GRB 211211A as a hybrid event, analogous to GRB 060614^3^. In addition to its prompt gamma-ray phase, several lines of evidence differentiate GRB 211211A from canonical long GRBs. The GRB does not lie in a star-forming region (Methods) and late-time optical imaging rules out any bright supernova at its location (Extended Data Fig. 4): as the dust content along the line of sight is negligible, a luminous supernova similar to SN 1998bw^15^ is excluded out to *z* ≈ 0.8. A faint and short-lived supernova similar to SN 2008ha^6^ is also ruled out by the optical limits. The GRB location and the global properties of its host galaxy provide indirect evidence for a stellar progenitor different from a collapsing massive star and are instead consistent with a compact binary merger (Methods).

The unambiguous proof of a compact object binary merger comes either from its gravitational wave signal^2^ or from its kilonova, a short-lived glow of quasi-thermal radiation powered by the radioactive decay energy of heavy nuclei^16^, produced in the merger ejecta via rapid neutron capture process (r-process). The first known kilonova was AT2017gfo, characterized by an early (less than 12 h) UV/optical peak^17^ followed by a longer-lasting infrared signal^18–21^. We find that a similar component is identified in the UV/optical/infrared (UVOIR) counterpart of GRB 211211A, providing us with the direct link to compact binary mergers.

**Fig. 2:**
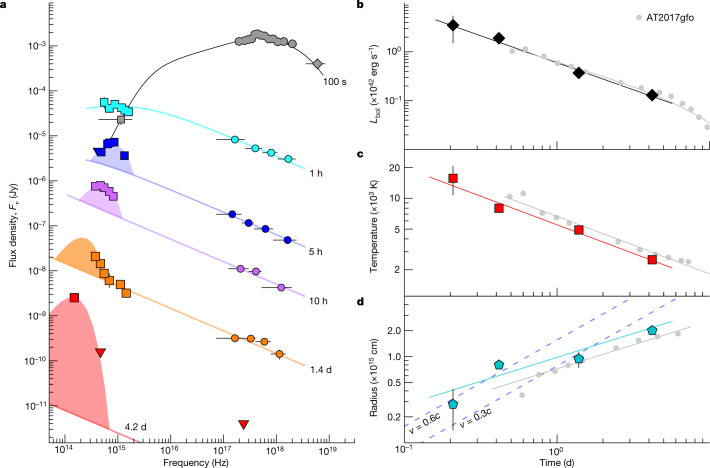
Spectral evolution of the GRB afterglow and kilonova. **a**, SED combining gamma-ray (diamonds), X-ray (circles) and UVOIR (squares) data at different times, as indicated by the labels. It shows that non-thermal radiation (solid line) dominates at early times and at higher energies. At lower energies, we identify the emergence of a thermal component peaking at blue wavelengths at 5 h, and rapidly shifting toward redder colours. Error bars represent 1*σ*; upper limits (downward triangles) are 3*σ*. For plotting purposes, each epoch was rescaled by the following factors (from top to bottom): 1, 1, 10^−0.8^, 10^−1.6^, 10^−2.4^, 10^−3.2^. **b**–**d**, The bolometric luminosity (**b**), temperature (**c**) and emitting radius (**d**) of the thermal component are similar to AT2017gfo^27^ (grey circles). Solid lines show the best-fit power-law models to the dataset. Dashed lines in **d** show the predicted radius for constant expansion velocities of 0.3*c* and 0.6*c* (*c*, speed of light in a vacuum).

The multiwavelength emission that follows a GRB is the superposition of multiple components. The dominant component is usually the afterglow, a broadband synchrotron radiation emitted by a population of electrons shock-accelerated by the GRB outflow^22^. We use the X-ray data to probe the contribution of this non-thermal component. The X-ray spectrum is well described by a power law with slope *β*_X_ ≈ 0.5 and negligible absorption along the line of sight. When extrapolated to lower energies, this model roughly matches the observed optical fluxes at *T*_0_ + 1 h and shows no evidence for an additional component at this time. However, at later times, the multifrequency spectral energy distribution (SED; Fig. 2) identifies emission in excess of the standard afterglow: the UVOIR counterpart is consistently brighter than the extrapolation of the non-thermal power-law, and is characterized by a steeper spectral index *β*_UVOIR_ > 2 for *t* > 1 d. Its spectral peak lies in the UV range (*u* band, observer frame) at *T*_0_ + 0.2 d and then progressively cools down to near-infrared wavelengths (*K* band at approximately *T*_0_ + 4 d).

**Extended Data Fig. 6:**
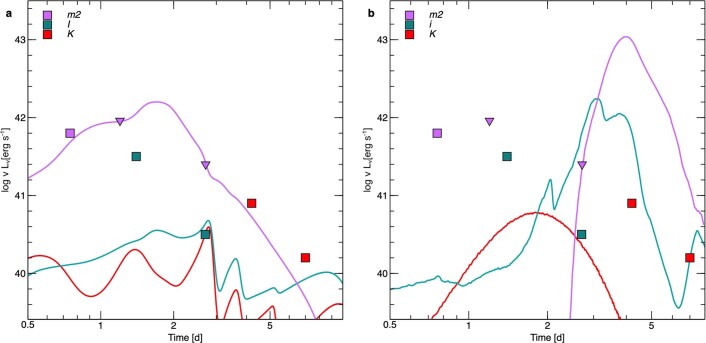
A comparison of models for luminous blue transients. **a**, **b**, The UVOIR counterpart (squares) is compared with a set of models producing luminous (*L*_bol_ ≈ 10^42^ erg s^−1^) and short-lived (<7 d) transients: a low-nickel SN from a fallback collapsar^25^ underpredicts the optical/near infrared emission (**a**); a magnetar-powered kilonova^31^ does not easily reproduce the timescales and colours (**b**). Upper limits (downward triangles) are 3*σ*.

We rule out that a reverse-shock-powered afterglow or a supernova onset could explain this low-energy component. The former arises within the GRB outflow and is characterized by an optical rebrightening peaking from a few seconds to approximately 1 h after the burst^23,24^. However, reverse-shock emission quickly cools off and shifts to the radio band, typically within the first day after the burst. This is not consistent with the observed SED evolution. Moreover, a low nickel-yield explosion would also produce a short-lived UV/optical flare powered by shock heating in the supernova blast wave^25^. We studied a broad range of collapsar-associated supernovae,  varying nickel yields, stellar properties and explosion energies. Although this model explains the lack of a bright supernova at late times and can reproduce the basic features of the early optical emission such as the bolometric luminosity and photospheric radii (Extended Data Table 1), the predicted spectrum is too hard (Extended Data Fig. 6): UV emission dominates and we cannot reproduce the bright and long-lived near-infrared emission without the addition of a second, neutron-rich outflow (see Methods).

After subtracting the afterglow contribution from the data, we find that the UVOIR excess is well described by a thermal spectrum and that the best-fit parameters point to a hot (*T* ≈ 16,000 K, rest frame) fireball in rapid expansion with apparent velocity *v* ≳ 0.5*c*. These properties do not match neither those of optical transients from white dwarf mergers (Supplementary Methods) nor those of a thermal dust echo^26^. Instead, the luminosity, temperature and emitting radius of this thermal component display a striking resemblance to AT2017gfo^27^ (Fig. 2), and we interpret it as the kilonova emission associated with GRB 211211A. A kilonova in GRB 211211A, and consequently its association with a compact binary merger, tie the lack of supernova, the GRB environment and the evolution of its UVOIR counterpart in a coherent explanation.

**Fig. 3:**
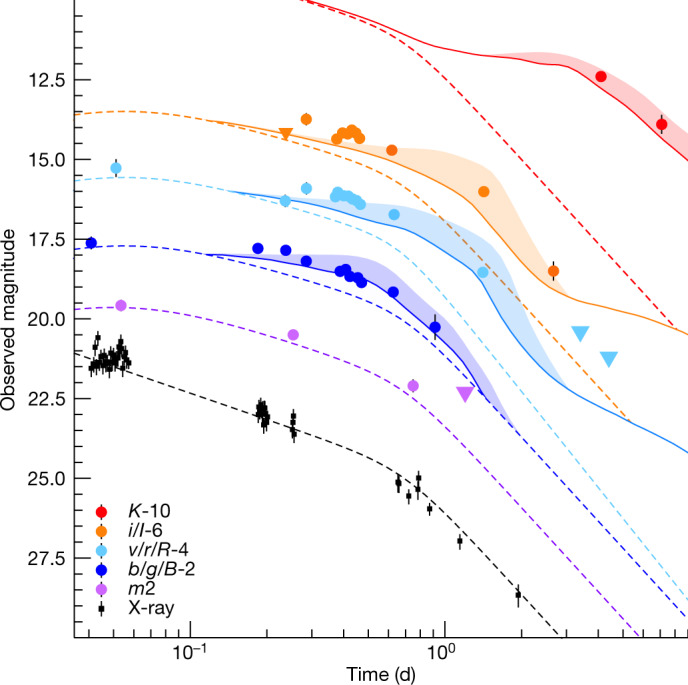
A kilonova in the long GRB 211211A. Multicolour light curves in X-ray, UV (*uvm*2), optical (*BRI*) and infrared (*K*) are compared to models’ predictions of a kilonova (solid line) in addition to the non-thermal emission (dashed line). The shaded area shows the range of possible fluxes reproduced by kilonova simulations with wind mass *M*_w_ between 0.01*M*_☉_ (lower bound) and 0.1*M*_☉_ (upper bound), and dynamical ejecta mass *M*_d_ between 0.01*M*_☉_ (lower bound) and 0.03*M*_☉_ (upper bound). Error bars represent 1*σ*; upper limits (downward triangles) are 3*σ*. For plotting purposes, light curves were shifted by a constant factor, as indicated by the numbers in the legend.

Our dataset allows us to probe the earliest phases of the kilonova onset, not observed in the case of AT2017gfo. Although the broadband emission is initially dominated by the non-thermal afterglow, evidence for a thermal component is found as early as *T*_0_ + 5 h. Figure 3 shows the different behaviours of the X-ray and UVOIR counterparts. The latter requires an additional component, which we model using simulated kilonova light curves^28^ with wind ejecta mass *M*_w_ in the range (0.01–0.1)*M*_⊙_, and dynamical ejecta mass *M*_d_ ≈ (0.01–0.03)*M*_⊙_ (*M*_⊙_, mass of the Sun). The ejecta velocity and kilonova bolometric luminosity, *L*_bol_ ≈ 3 × 10^42^ erg s^−1^ (isotropic equivalent) inferred at early times, are challenging to reproduce with purely radioactive-powered models^28^, even when accounting for different density profiles and the larger projected area along the polar axis^29^ (Methods). We therefore explore alternative models in which the merger ejecta is re-energized by a central engine or modified by the interaction with the GRB jet. The former group of models, envisioning either a highly magnetized neutron star or fallback accretion onto the central black hole, is often invoked to explain a long-lasting gamma-ray emission^30,31^. However, an active engine would leave observable imprints on the kilonova light^32^, which are not consistent with its timescales (too early) or colours (too red) (Extended Data Fig. 6).

We therefore consider a model in which jet–ejecta interactions shape the observed emission. A relativistic jet is present in both GRB 211211A and GRB 170817A and its effects may explain their similar kilonova evolution. As the jet propagates through the massive (≳0.01*M*_⊙_) cloud of radioactive ejecta, it heats and partially disrupts its density structure, carving a funnel of low-opacity, low-density material along the polar axis^32^. By exposing the inner, hotter surface of the ejecta, an energetic (*E*_γ,iso_ ≈ 6 × 10^51^ erg) GRB jet makes the kilonova emission both bluer and brighter^33^ for an observer close to its axis. Shock heating may also contribute to distribute the energy. Viceversa, the ejecta imparts a wide angular structure on the GRB jet before it breaks out^34,35^. High-latitude emission from the jet wings arrives later because of the longer path that the photons travel and may produce a low-luminosity, fast-fading X-ray transient^36^ consistent with the observed X-ray behaviour. This feature may become visible in the case of a ‘naked’ structured GRB jet expanding into a low-density circumburst medium such as GRB 211211A.

We conclude that, although the long duration of the prompt phase challenges our understanding of compact binary merger models, a merger progenitor naturally explains all the other observed features of GRB 211211A. At 346 Mpc, this GRB lies within the distance horizon of forthcoming gravitational wave observing runs^37^ and, had the gravitational wave network been online at the time of the burst, this event would have probably resulted in a joint detection of gravitational waves and electromagnetic radiation. We note that some of its electromagnetic properties are very different from the multimessenger transient GW170817; whereas the electromagnetic counterparts of GW170817^2,17,20^ would be challenging to localize beyond approximately 150 Mpc, GRB 211211A would be visible out to *z* ≈ 1 by most space-borne gamma-ray detectors. Moreover, rapid X-ray and UV/optical follow-up would detect its counterpart out to *z* ≈ 0.2 assuming a sensitivity comparable to Swift.

**Extended Data Fig. 7:**
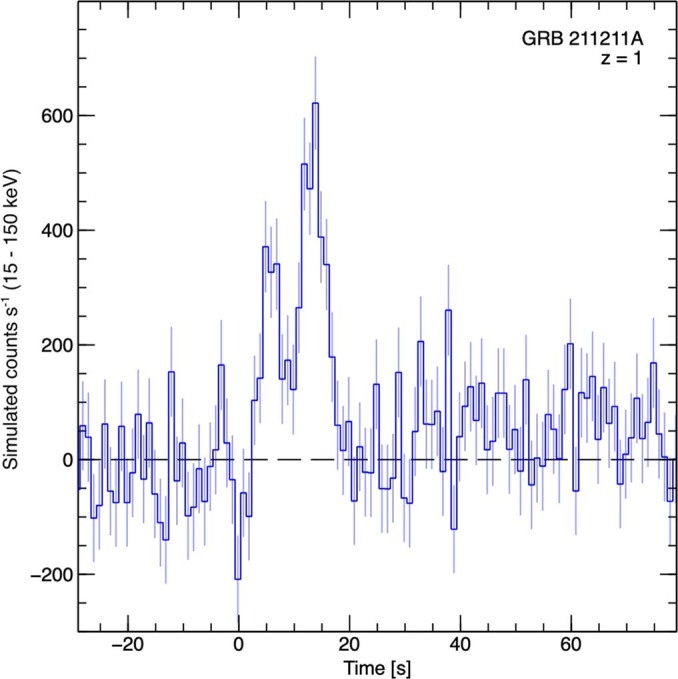
GRB 211211A at high redshift. Simulated Swift light curve in the 15–150 keV energy band (observer’s frame) of GRB 211211A assuming *z* = 1, an incident angle of 45°, and ~6,000 counts s^−1^ background rate. Time bin is 1 s, error bars represent 1*σ*. An event similar to GRB 211211A would be detected up to redshift *z* ≈ 1 and beyond. At these distances, it would appear as a standard long GRB with a duration of ~20 s.

To determine the rate of hybrid GRBs, we examine the Swift GRB catalogue^11^ in search of bursts similar to GRB 211211A and GRB 060614. At large distances (*z* ≈ 1), their classification would rely solely on the high-energy properties, which point to regular bursts of long duration (Extended Data Fig. 7). Without a systematic study of GRB lags, spectra and durations it is not possible to assess the total number of hybrid bursts detected thus far. Therefore, we turn to lower redshifts where a clear observational signature of these events is the lack of a supernova. Supernovae associated with GRBs^1^ peak between *M*_V_ ≈ −18.5 mag and *M*_V_ ≈ −20 mag, and sensitive supernova searches are regularly undertaken for GRBs within a redshift *z* < 0.3, which we identify as the maximum distance for a homogeneous identification. We review the entire sample of Swift bursts with duration greater than 2 s and a putative host galaxy at *z* < 0.3 and find a total of 20 GRBs in 17 years of mission (2005–2021). Of these, eight are associated with a supernova, three have no meaningful constraints, and nine have deep limits on any accompanying supernova. The chance alignment between a bright galaxy and an afterglow with subarcsecond localization is typically less than 1% (ref. ^11^), thus it is unlikely that all nine bursts are distant background objects. Furthermore, four of them (GRB 060614, GRB 060505, GRB 191019A and GRB 211211A) have UV counterparts constraining their distance scale^5,38,39^. We conclude that some of these long-duration bursts are physically associated with a low-redshift galaxy and lack a supernova, forming a new class of hybrid GRBs produced by compact binary mergers. After accounting for instrumental effects (Supplementary Methods), we derive a volumetric all-sky rate of 0.04–0.8 Gpc^−3^ yr^−1^ (68% CL), lower than the observed rate of short GRBs^40^. The true rate of events depends on the unknown beaming factor *f*_b_ of these outflows. Assuming similar jet properties to short GRBs^41^, hybrid long-duration bursts may represent approximately 10% (0.8%–26%, 68% CL) *f*_b,short_/*f*_b,hybrid_ of the population of electromagnetic counterparts to gravitational wave sources caused by compact binary mergers.

## Methods

### Classification of GRB 211211A

GRBs are classified based on the properties of their prompt gamma-ray phase. The prompt emission of GRB 211211A (Extended Data Fig. 1) displays three different episodes: a weak precursor, a bright multipeaked main burst and a highly variable temporally extended emission. The time intervals for spectral and temporal analysis were selected to characterize them separately. Swift and Fermi data were processed using HEASOFT v.6.30. Spectra were extracted from the Fermi Gamma-ray Burst Monitor data and fitted within XSPEC^42^. The temporal properties were derived from the Swift BAT light curves using well-established techniques^43,44^.

The precursor phase has a short duration of 0.15 s, a soft spectrum peaking at ~75 keV, a minimum variability timescale of 21 ± 4 ms, and a positive lag ${\tau }_{31}={16}_{-3}^{+4}\;{\rm{ms}}$ (68% CL; uncertainties throughout are quoted at 68% CL unless otherwise stated) between the temporal structures observed in the 50–100 keV (band 3) and in the 15–25 keV (band 1) energy bands, respectively. At 346 Mpc, the measured flux of 8 × 10^−7^ erg cm^−2^ s^−1^ (10–1,000 keV) corresponds to a luminosity of only ~10^49^ erg s^−1^.

After a 1-s period of quiescence, we detect the onset of the main prompt emission, which consists of multiple overlapping peaks lasting for approximately 10 s. The time-averaged spectrum peaks at 750 ± 10 keV, the minimum variability timescale is 14 ± 5 ms, and the temporal lag is negligible with ${\tau }_{31}=-{0.9}_{-2.6}^{+2.8}\;{\rm{ms}}$. The total fluence measured during this episode is ~3.7 × 10^−4^ erg cm^−2^ (10–1,000 keV), one of the highest ever measured for a GRB. However, at 346 Mpc the total isotropic-equivalent gamma-ray energy *E*_γ,iso_ would be ~5 × 10^51^ erg within the typical GRB range^45^.

A brief (3-s) period of low-level persistent emission precedes the onset of a long-lasting tail. The time-averaged spectrum of the extended emission has a soft peak of 52 ± 2 keV, the minimum variability timescale is 42 ± 9 ms, and the lag, ${\tau }_{31}={7}_{-2}^{+3}\;{\rm{ms}}$, is positive. The total fluence is ~5 × 10^−5^ erg cm^−2^ (10–1,000 keV), corresponding to *E*_γ,iso_ ≈ 7 × 10^50^ erg.

We compare the properties of the main prompt emission to the population of GRBs using four classifiers: the duration/hardness-ratio diagram^46^, the lag–luminosity relation^43^, the variability timescale^44^ and the Amati correlation^45^ (Extended Data Fig. 2). Similar to GRB 060614^3^, GRB 211211A shows characteristics that are intermediate between the two main GRB classes: the traditional classification based on duration and hardness ratio places this event in the class of long GRBs; however, its other properties fit within the class of short bursts. Its hybrid nature does not allow us to unambiguously link it to a progenitor system solely on the basis of its high-energy properties.

### The GRB environment and its host galaxy

The GRB environment typically offers stringent, albeit indirect, evidence of its progenitor system. In the case of GRB 211211A, no underlying host galaxy is detected in late-time HST imaging (Fig. 1). By planting artificial sources with an exponential disk profile and different brightness, we derive an upper limit of F814W > 26.5 AB mag and F160W > 27.6 AB mag. Because no coincident galaxy is found, we analyse the GRB field to search for its most probable host. We identify seven galaxies within 10″ from the GRB position (Fig. 1): G1 with *r* = 19.50± 0.02 mag at an offset of 5.55″ ± 0.03″, G2 with *r* = 20.88 ± 0.05 mag at an offset of ~10″, and five faint (*r* > 26 AB mag) extended objects at an offset between 2.5″ and 10″. By using the galaxy’s number counts in the *r*-band^47^, we derive a chance alignment *P*_cc_ of 1.4% for G1, >10% for G2, and >40% for the other faint galaxies. Therefore, probabilistic arguments favour the association between GRB 211211A and G1. We note that the probability threshold adopted to associate a galaxy with a GRB is generally >1%, meaning that G1 with *P*_cc_ ≈ 1.4% would be considered as the most probable host by any previous studies of GRB galaxies^12,47^. Moreover, in our spectroscopic observations we find no evidence for any emission lines at the GRB position down to >2 × 10^−17^ erg cm^−2^ s^−1^ Å^−1^ in the range 4,800–6,100 Å. Using [O ii] 3727 and Hβ as indicators of unobscured star formation^48^, we place an upper limit on the star-formation rate, SFR < 1*M*_⊙_ yr^−1^ for *z* < 0.65. This corresponds to the median SFR of long GRB hosts^49^ at *z* < 1, providing additional constraints on any possible underlying galaxy.

The spectrum of G1 shows several emission lines including Hα, [N ii], and [S ii] at a common redshift of *z* = 0.0762 ± 0.0003, consistent with a previous report^9^ based on data from the Nordic Optical Telescope (NOT). Assuming a *Λ*CDM cosmology^50^ with a Hubble constant of *H*_0_ = 69.8 km Mpc^−1^ s^−1^, we find a luminosity distance *d*_*L*_ = 346 Mpc, and a distance modulus *μ* = −37.7 mag. Using the host galaxy photometry (Supplementary Table 1), we compute a rest-frame absolute *B*-band magnitude of *M*_*B*_ ≈ −17.6 AB mag, corresponding to *L*_*B*_ ≈ 0.1*L*⁎ (*L*⁎, characteristic luminosity of the Schechter function) when compared to the galaxy luminosity function^51^ at a similar redshift (0.05 < *z* < 0.2).

The brightness (*L*_Hα_ ≈ 10^40^ erg s^−1^) and relative ratio of these lines (log([N ii]/Hα) ≈ −0.7) point to a star-forming galaxy with SFR ≈ 0.05*M*_⊙_ yr^−1^ and sub-solar metallicity 12 + log(O/H) ≈ 8.4. We also find evidence for weak [Mg i *λ*5175Å] absorption at ~5,567 Å, indicative of an evolved stellar population, although this feature is affected by a nearby skyline.

We model the galaxy’s surface brightness using GALFIT^52^. A good description (${\chi }_{\nu }^{2}\approx 1.03$) of its morphology is obtained by including two Sersic profiles with index *n* = 1, one with half-light radius *R*_e,1_ ≈ 2.15 arcsec (F814W; ~3.1 kpc at *z* = 0.076) and one with *R*_e,2_ ≈ 0.5 arcsec (F814W; ~0.7 kpc at *z* = 0.076) to model the central bar. Similar results are obtained on the F160W image with *R*_e,1_ ≈ 2.34 arcsec and *R*_e,2_ ≈ 0.64 arcsec. The half-light radius *r*_50_ ≈ 1.1 arcsec obtained through Source Extractor is given by the weighted average of these two components.

The galaxy’s global properties were determined by modelling its SED (Supplementary Table 1) with Prospector^53^, adopting the same settings used for GRB host galaxies^12,54^. We derived a stellar mass of $M={0.9}_{-0.4}^{+0.2}\times 1{0}^{9}{M}_{\odot }$, a star-formation rate SFR = (0.06 ± 0.02)*M*_⊙_ yr^−1^, a low dust content ${A}_{V}={0.09}_{-0.06}^{+0.08}\;{\rm{mag}}$, and a mass-weighted stellar age $\tau ={5}_{-3}^{+2}\;{\rm{Gyr}}$. When compared to the sample of long GRBs, the properties of the host of GRB 211211A are not unprecedented but extremely uncommon. The inferred SFR lies in the bottom 10% of the observed distribution, leading to an unusually low specific SFR, sSFR ≈ 0.06 Gyr^−1^. This value is below the main sequence of star-forming galaxies^55^, indicating that G1 may be migrating to a quiescent phase. This differs from the typical environment of long GRBs at both high and low redshifts: for comparison, nearby events such as GRB 060218 and GRB 100316D were associated with sSFR ≈ 4 Gyr^−1^ and sSFR ≈ 0.2 Gyr^−1^, respectively^56,57^. Dissimilarities with the class of short GRBs also exist: the stellar mass lies at the bottom 10% of both short GRB and supernova type-Ia host galaxies^58,59^, as for GRB 060614, which was hosted by a dwarf galaxy^5^.

### SED

The SED of the GRB counterpart at different times is shown in Fig. 2. These epochs were selected to maximize simultaneous multiwavelength coverage. When needed, the data were rescaled to a common epoch using the best-fit temporal model.

In the first epoch at *T*_0_ + 100 s, the X-ray emission is characterized by a flat spectral index *β*_X_ = 0.00 ± 0.03. A spectral break is required above ~10 keV to account for the lower flux and soft spectral index, *β*_BAT_ ≈ 2, measured in the hard X-ray band. In addition, the high X-ray-to-optical flux ratio, *F*_X_/*F*_O_ ≈ 100, requires a turn-over to a steep spectrum between the X-ray and optical band. These properties are consistent with self-absorbed synchrotron radiation in the fast-cooling regime. The location of a self-absorption frequency, *ν*_a_ ≈ 10 eV, indicates a compact emitting region^60^ with radius *R* ≈ 10^13^(*Γ*/300)^3/4^ cm, where *Γ* is the outflow bulk Lorentz factor. This radius is typical of dissipation processes within the GRB outflow, indicating that at ~*T*_0_ + 100 s the prompt phase is still dominant at both X-ray and optical wavelengths.

In the second epoch at *T*_0_ + 1 h, the GRB counterpart displays blue colours with a spectral index *β*_O_ = 0.23 ± 0.10 in the UV and optical bands. At X-ray energies the spectrum, extracted between 3 ks and 5 ks, has a slope of *β*_X_ = 0.50 ± 0.05. This index points to synchrotron radiation in the slow cooling regime, in which the cooling frequency is *ν*_c_ > 10 keV and the synchrotron frequency is *ν*_m_ ≲ 1 eV. In this case, the X-ray spectral slope is related to the energy distribution of the emitting electrons, *N*(*E*) ∝  *E*^−*p*^ with *p* = 2*β*_X_ + 1 = 2.0 ± 0.1. This is a fundamental constraint to the long-term afterglow evolution. The steepest spectral slope explained by this model is *p*/2 ≈ 1.05, and only for energies above *ν*_c_. Therefore, the UVOIR and X-ray non-thermal afterglows are bound to remain on the same spectral segment over the time span of our observations.

Starting from ~*T*_0_ + 5 h, a simple non-thermal spectrum can no longer reproduce the broadband emission. An UVOIR excess is detected at all epochs. It is characterized by a narrow spectral shape peaking in the *u* band, well described by a blackbody function with temperature *T* ≈ 16,000 K (rest frame) and a luminosity *L*_bol_ ≈ (3.5 ± 2.0) × 10^42^ erg s^−1^. We therefore fit each SED epoch with a blackbody (UVOIR) plus power-law (X-ray) model, and derive the total integrated blackbody luminosity, its temperature and radius as a function of time (Fig. 2 and Extended Data Table 1). The luminosity is better constrained in our second epoch at *T*_0_ + 10 h, *L*_bol_ = (1.90 ± 0.15) × 10^42^ erg s^−1^ and is seen to decrease in time following a power-law ∝*t*^−0.95^, consistent with the evolution of AT2017gfo^27^.

### GRB distance scale

We investigate the joint X-ray/UV/optical SED at 1 h to place a direct upper limit on the GRB distance scale. UVOT spectra were created with the tool uvot2pha using the same source and background regions selected for photometry. We adopt a power-law model and include the effects of absorption, dust reddening and intergalactic medium attenuation as implemented in the XSPEC models zphabs, zdust and zigm. The Galactic absorption was fixed to *N*_H_ = 1.76 × 10^20^ cm^−2^ and the reddening at *E*(*B* − *V*) = 0.015 mag. All other parameters were left free to vary. We increase the redshift from 0 to 2.5 in steps of 0.1 and find the best-fit model by minimizing the Cash statistics, recording its value at each step. On the basis of the variations of the test statistics, we derive an upper limit of *z* < 2.3 (99.9% CL) from the UV/optical data, and *z* < 1.5 (99.9% CL) from the joint X-ray/UV/optical fit. By imposing the redshift of the putative host galaxy, *z* ≈ 0.0762, we find no evidence for any dust extinction or absorption at the GRB site with 3*σ* upper limits of *E*(*B* − *V*)_*z*_ < 0.005 mag and *N*_H,*z*_ < 9 × 10^19^ cm^−2^, respectively. This is consistent with the location of the GRB, well outside the galaxy’s light.

### Origin of the X-ray afterglow

Swift observations show a rapidly fading X-ray afterglow followed by a shallower decline *F*_X_ ∝ *t*^−*α*^ with $\alpha ={1.11}_{-0.07}^{+0.08}$ between 1 ks and 40 ks, and a final steep decay with *α* = 3 ± 0.5 after 40 ks. On the basis of this model, we infer an X-ray flux of ~4 × 10^−12^ erg cm^−2^ s^−1^ at 11 h. This corresponds to a luminosity *L*_X_ ≈ 6 × 10^43^ erg s^−1^ at 346 Mpc, nearly two orders of magnitude below the typical X-ray luminosity of cosmological GRB afterglows at this epoch (see figure 7 of ref. ^23^). The low ratio between the observed X-ray flux and the emitted gamma-ray fluence, log*f*_X,11hr_/*F*_γ_ ≈ −7.9, is indicative of atypical properties for this explosion (compare with figure 17 of ref. ^12^).

Our SED analysis (Fig. 2) demonstrates that the X-ray counterpart is dominated by non-thermal emission consistent with synchrotron radiation. Although we interpret the early (<300 s) X-ray emission as the tail of the prompt phase, at later times (>1,000 s) the most common origin of non-thermal afterglow radiation is the interaction between the ambient medium and the GRB jet occurring at large distances (>10^17^ cm) from the central source. In this external-shock model^61^, a flux decay rate of 2 or faster is explained by geometrical factors owing to the collimation of the GRB outflow^62^. The time *t*_j_ at which the light curve steepens, the so-called jet break, increases with the jet opening angle *θ*_c_. A jet break at 40 ks would require a very narrow jet, and then can only achieve a decay of *α* = *p* ≈ 2.1, in mild tension with the observations. We tested the hypothesis of an early jet break by modelling the X-ray and early (~*T*_0_ + 1 h) UVOT data with afterglowpy^63^ assuming a uniform external environment and both a top-hat and a Gaussian lateral structure for the jet. Despite the dataset being limited, it provides tight constraints to the model: the flat UVOT SED at *T*_0_ + 1 h (Fig. 2) requires the synchrotron peak to lie close to the optical range, constraining the value of the synchrotron frequency *ν*_m_ and the peak flux *F*_pk_; the X-ray spectrum places the cooling frequency at *ν*_c_ > 10 keV and provides a measurement of *p* ≈ 2.0–2.1, and the X-ray light curve constrains the jet opening angle *θ*_c_ and the viewing angle *θ*_v_. We performed Bayesian parameter estimation with emcee^64^ and nine free parameters: *n*, *p*, *E*_K,iso_, *θ*_c_, *θ*_v_, an outer jet truncation angle *θ*_w_, shock microphysical parameters *ε*_e_ and *ε*_B_, and the participation fraction *ξ*_N_. The best fit has a reduced chi-squared ${\chi }_{\nu }^{2}\approx 1.8$; fits with *ξ*_N_ frozen at 1 found a similar ${\chi }_{\nu }^{2}$ but required unphysical shock parameters *ε*_e_ ≈ *ε*_B_ ≈ 1. The parameter estimation reports a jet of energy *E*_K,iso_ ≈ (0.8–17) × 10^51^ erg, width *θ*_c_ ≈ 1.9–5.7°, viewed at *θ*_v_ ≈ 1.1–5.4° from the jet axis. The external density is *n* ≈ 0.016–12 cm^−3^. The shock parameters are *p* ≈ 2.1–2.2, *ε*_e_ ≈ 0.05–0.77, *ε*_B_ ≈ (0.1–6.0) × 10^−4^, and *ξ*_N_ ≈ (0.8–9.6) × 10^−2^. The beaming-corrected kinetic energy of the jet in this scenario is (0.4–4.4) × 10^49^ erg. Assuming that the angular size corrections between the afterglow and prompt emissions are similar, this scenario gives ~65% probability to an unphysical gamma-ray efficiency *η*_γ_ = *E*_γ,iso_/*E*_K,iso_ > 100% and a 90% probability *η*_γ_ > 15%. In combination with the poor reduced chi-squared of 1.8 we conclude it is challenging for an external shock to simultaneously reproduce the salient features of the GRB afterglow—a flat UV/optical spectrum at *T*_0_ + 1 h, an X-ray spectrum *β*_X_ ≈ 0.5, and a steep decay of the X-ray flux after 40 ks—while remaining within the energetic limits of the prompt emission. This tension may be alleviated when considering the effects of inverse Compton cooling. In the limit of Thompson-scattering-dominated inverse Compton cooling^65^, we estimate that the required isotropic energy would increase by a factor of ~100, and the density decreased by a factor of ~1,000. However, the jet opening and viewing angles would have to decrease down to 0.5° to reproduce the final steep decay.

If not caused by a jet break, a rapid drop in brightness is difficult to produce, owing to the relativistic and extended nature of the GRB outflow. Owing to the curvature effect^13^, any rapid decrease in brightness in the lab frame of the GRB will be smeared out in the observer frame as a result of the different arrival times of the photons, producing a decay of *α* = 2 + *β*_X_ ≈ 2.5. Nevertheless, this is a steeper slope than that allowed by the jet-break model and may present a better description than the standard external shock. If interpreted as a curvature effect, the steepening at 0.5 d links the observed X-ray emission either to long-lasting activity of the central engine, as in the ‘internal plateau’ model^66,67^, or to the angular structure of the GRB jet. If a structured jet produces GRB prompt emission in the high-latitude regions (the jet ‘wings’), this emission would be Lorentz-deboosted relative to the core prompt emission and delayed via the curvature effect^36^. With appropriate jet structures, this can manifest as X-ray emission with a shallow decay followed by a steep declining light curve. This feature, normally hidden by the brighter external shock emission, may become apparent in the case of a ‘naked’ structured GRB exploding in a rarefied medium. This latter model offers a consistent explanation of the X-ray behaviour of GRB 211211A and its physical offset from the galaxy without the requirement of hours-long activity of the central engine.

Despite uncertainty in the physical origin of the afterglow emission, the observed X-ray spectrum is well measured and its extrapolation to the UVOIR bands unambiguously places it below the UV/optical detections after ~*T*_0_ + 5 h. The observed UVOIR excess was measured by subtracting this extrapolated non-thermal component. This procedure does not require a physical interpretation of the non-thermal emission and provides an upper bound on the non-thermal contribution in the UVOIR bands. Thus the identification of the UVOIR excess does not depend on the specific physical interpretation of GRB 211211A’s non-thermal emission.

### Origin of the UVOIR excess

#### Collapsar model

We first examine the most common case of a long GRB produced by the collapse of a rapidly rotating massive star (collapsar). The emergence of the supernova blast wave can produce a luminous blue emission in excess of the standard afterglow^25^, and we test whether this is consistent with the observed UVOIR excess in GRB 211211A. Collapsars arise from compact stellar cores and produce energetic and long-lived type-Ic supernovae or hypernovae. However, if the collapsar engine does not produce considerable ^56^Ni (for example, from a fallback collapsar), the blast wave produces a short-lived supernova light curve that dies out in the first 10 d. To test this model, we ran a series of hypernova explosions, varying the mass ((2.5–40)*M*_⊙_) and density profile (varying the slope in the density of the core and envelope) of the progenitor star as well as the explosion energy (spherically 10^51^–10^52^ erg). Although we can reproduce the evolution of the bolometric luminosity (Extended Data Table 1), the early-time emission in our best-fit model is too energetic (in the UV and extreme UV). As the ejecta cools, the emission peaks in the infrared at late times, but the luminosity is several orders of magnitude too dim to explain the observations. To account for the optical and infrared emission, the photosphere of the rapidly expanding supernova must uncover the collapsar accretion disk and wind ejecta from this disk must have similar-enough properties to neutron star merger disks^68,69^ to produce a kilonova-like transient. However, even in this case, the large mass reservoir of a collapsar would power a long-lived late-peaking transient, not consistent with the observations.

For the collapsar model to work, we must also explain the offset of the GRB from its host galaxy. O/B stars in binaries can be unbound during the supernova explosion of the primary star, imparting a ‘kick’ of up to 200 km s^−1^ onto the O/B companion^70^. This proper motion could move the companion O star well beyond its star forming region (~1 kpc in 5 Myr), but it is unlikely that this kick is sufficient to explain the large offset of this burst. In summary, a massive star progenitor for GRB 211211A would naturally account for its long duration but requires a combination of unusual circumstances (a low ^56^Ni yield explosion, a low-mass neutron-rich disk outflow, and an extreme kick velocity) to explain the entire set of observations.

#### Compact binary merger model

The observed excess emission is much better fit by the ejecta from a compact binary merger, composed either of two neutron stars or a neutron star and a stellar mass black hole. Figure 3 shows the range of model predictions consistent with the observations: only a small subset of light curves (4 out of 900 in the ‘on-axis’ angular bin; *θ*_v_ ≈ 0–16°) match the observing constraints. The near-infrared luminosities are well described by dynamical ejecta of mass *M*_d_ ≈ (0.01–0.03)*M*_⊙_, lower than the value inferred for GRB 060614^7,8^. The bright UV/optical counterpart suggests a massive (>0.01*M*_⊙_) wind component to the kilonova ejecta. However, the time-dependent spectra from the Los Alamos National Laboratory (LANL) grid of kilonova models^28^ produce light curves that are too dim to match the observed UV/optical luminosities or require too large an ejecta mass (~0.1*M*_⊙_). Models with large ejecta mass (*M*_w_ ≈ 0.1*M*_⊙_) better fit the early time data but overpredict the fluxes at later times; by contrast, the model with lower ejecta mass (*M*_w_ ≈ 0.01*M*_⊙_) provides a good description of the dataset only after ~11 h. All consistent models adopt a toroidal morphology for the high-opacity ejecta and a polar outflow of low-opacity ejecta and high expansion velocity *v*_w_ ≈ 0.3*c*.

It is probable that a number of alterations to the kilonova ejecta mechanism can help explain the early excess emission. For example, we have not conducted a detailed study varying the composition that changes both the opacity and the radioactive heating. Uncertainties in radioactive energy deposition^71^ and in the properties of the disk-wind ejecta allow for a wide range of behaviours and our study here only touches the surface of all possibilities. However, in its simplest form, a radioactive-powered kilonova captures the late-time evolution of the observed UVOIR transient but has difficulties in reproducing the bright optical emission seen at early times (*T*_0_ + 0.2 d).

An alternative way to alleviate the requirement on the ejecta mass is that the kilonova is powered by an additional energy source or affected by the jet–ejecta interactions^33^. To study the engine-powered models, we used the same method as in previous studies^31^. For central power sources—either a magnetar or fallback accretion on the central black hole—the energy must transport out from the centre to affect the light curves. In these models^31^, the central power sources do not alter the emission until ~5 d after the merger for wind mass ~0.01*M*_⊙_. However, if the jet is able to evacuate a region above the compact remnant, this delay can be reduced. We mimicked this evacuation by a series of spherically symmetric models, reducing the total wind mass to ~10^−7^*M*_⊙_. Although the signal peaks earlier it is still too late to explain our observations and the resultant spectrum is too high energy (peaking in the extreme UV; Extended Data Fig. 6). Turbulent motion may help to accelerate the UV peak by advecting the energy toward the outer layers more rapidly.

Although we caution that kilonova models are affected by large systematic uncertainties, we find that the majority of engine-driven kilonova models^31,72,73^ peak several hours or days after the merger, whereas jet–ejecta interactions remain a plausible solution to enhance the early emission.

In summary, we find that a compact binary merger would naturally account for most of the observed features of GRB 211211A, from the onset of its kilonova to its environment and high-energy properties. The main challenge to this model remains the long duration of the prompt gamma-ray emission, requiring an active central engine for up to ~100 s.

## Online content

Any methods, additional references, Nature Research reporting summaries, source data, extended data, supplementary information, acknowledgements, peer review information; details of author contributions and competing interests; and statements of data and code availability are available at 10.1038/s41586-022-05327-3.

### Supplementary information

Supplementary Methods This file includes a description of the X-ray, ultraviolet, optical and infrared observations, their reduction and analysis. We discuss models for white dwarf mergers, and derive the rate of events similar to GRB 211211A. A table reporting the photometry of the GRB counterpart and its host galaxy is available.

## Figures and Tables

**Extended Data Fig. 3:**
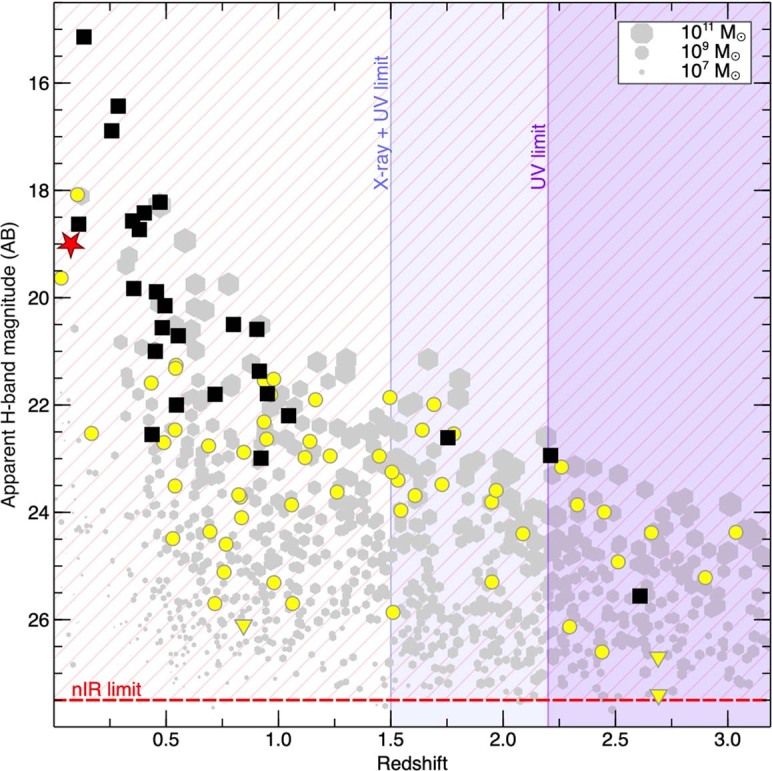
Constraints on the distance scale of GRB 211211A. The near infrared brightness of GRB host galaxies (short GRBs: squares; long GRBs: circles) is reported as a function of their redshift. For comparison, a randomly selected sample of field galaxies from the CANDELS survey^74^ is shown in the background (octagons, with the symbol size proportional to the galaxy mass). The non-detection of an underlying galaxy in deep HST F160W imaging rules out most of the parameter space occupied by GRB hosts (hatched area). Additional constraints from the UV and X-ray afterglow rule out the case of a GRB in a distant (*z* > 1.5; shaded areas) faint galaxy. These observations support the physical association between GRB 211211A and the nearby galaxy at *z* = 0.0762 (star symbol). Upper limits (downward triangles) are 3*σ*.

**Extended Data Fig. 5:**
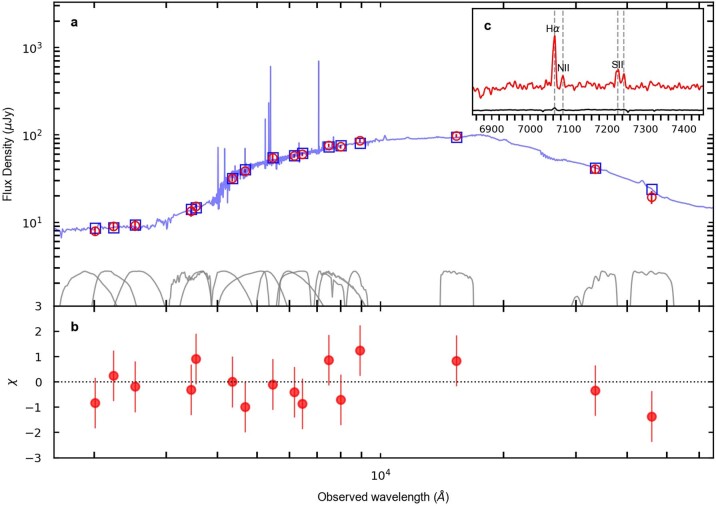
Host galaxy spectral energy distribution. **a**–**c**, The model SED (blue line) and model photometry (blue squares) derived using Prospector is compared to the observed photometry (red circles) (**a**). The fit residuals (**b**) are displayed in the bottom panel. The inset (**c**) displays a Gemini/GMOS-S spectrum of the host galaxy in the vicinity of H*α*, [N ii], and the [S ii] doublet, yielding *z* ≈ 0.0762. Error bars represent 1*σ*.

**Extended Data Table 1:**
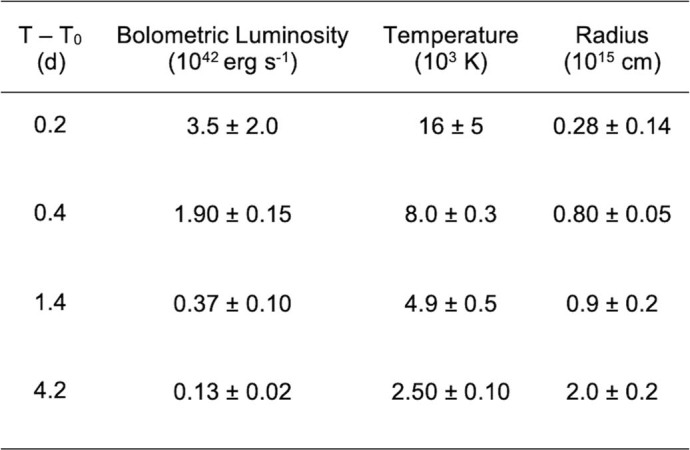
Best-fit blackbody parameters

Temporal evolution of the kilonova properties estimated from blackbody fitting. Errors are 1*σ*.

## Data Availability

Data from NASA’s missions are publicly available from the High Energy Astrophysics Science Archive Research Center (HEASARC) at https://heasarc.gsfc.nasa.gov. Swift XRT products are available from the online GRB repository https://www.swift.ac.uk/xrt_products. Other data are available from the corresponding author upon reasonable request. The broad grid of kilonova models is publicly available at 10.5281/zenodo.5745556.

## Code availability

Results can be reproduced using standard free analysis packages. Methods are fully described. Code used to produce figures can be made available upon request.
