# Supplementary material for: A nearby long gamma-ray burst from a merger of compact objects
Source: Nature. 2022 Dec 7;612(7939):228–31. doi: 10.1038/s41586-022-05327-3 (PMC9729102; doi:10.1038/s41586-022-05327-3)
Supplement: Supplementary file 1 — This file includes a description of the X-ray, ultraviolet, optical and infrared observations, their reduction and analysis. We discuss models for white dwarf mergers, and derive the rate of events similar to GRB 211211A. A table reporting the photometry of the GRB counterpart and its host galaxy is available. [file 41586_2022_5327_MOESM1_ESM.pdf]

nature portfolio

https://doi.org/10.1038/s41586-022-05327-3

Supplementary information

A nearby long gamma-ray burst from a merger of compact objects

In the format provided by the authors and unedited

Supplementary Methods

Observations and Data Reduction

Swift - The *Swift* X-ray Telescope (XRT) began observations of GRB211211A at 69 s after the GRB trigger and continued monitoring for ~3 days. The X-ray lightcurve and spectra were retrieved from the *Swift* XRT GRB repository[77]. The GRB counterpart was detected by the UltraViolet and Optical Telescope (UVOT) in all filters. UVOT data were analyzed using HEASOFT v.6.30. Individual exposures from the same epoch were aligned and then combined using the task *uvotimsum*. To minimize the contamination from the nearby galaxy G1, we determined the source count-rates in 3" circular apertures with the background estimated in a concentric source-free annular region. We used an aperture correction to determine the equivalent source count-rates in a 5" aperture, and then applied standard UVOT magnitude zeropoints[78]. Late-time images were used to determine the host galaxy brightness using a 5" circular aperture. The photometry of the transient and its host galaxy is reported in Supplementary Table 1.

Optical/nIR imaging - Our observations of GRB211211A began at T_0+5.1 hr with the 50-cm Multicolor Imaging Telescopes for Survey and Monstrous Explosions (MITSUME[79,80,81]) acquiring 110×60 s simultaneous images in *g*, R_c, and I_c filters. Observations were carried out for three consecutive nights, but the GRB counterpart was only detected on the first night. The data were reduced using the MITSUME pipeline[82] and image subtraction between the first, second and third epoch was performed using HOTPANTS[83]. Upper limits were derived by planting artificial sources of known brightness, then repeating the image subtraction step.

Starting on 2021 Dec 11 UT 22:11:29 (~T_0 + 9.0 hr), multi-band (*U, B, V, R, I*) optical observations of GRB211211A were carried out using the 4Kx4K CCD Imager mounted at the axial port of the 3.6m Devasthal Optical Telescope[84,85,86,87] (DOT). Observations continued until twilight time (~T_0 + 11.4 hr) and were repeated in subsequent nights until the optical

counterpart was no longer detected. Starting on 12 Dec 2021 at 3:53 UT (T_0 + 15 hr), additional imaging of the GRB field was carried out with the 2.2m telescope, equipped with the Calar Alto Faint Object Spectrograph (CAFOS), at the Calar Alto Observatory in Almería (Spain) using the $g'r'i'$ Sloan filters.

Additional epochs of observations were performed between 2021 Dec 31 and 2022 Jan 02 to search for the possible SN peak. We acquired multiple R-band images (24×300s) with the 1.3m Devasthal Fast Optical Telescope (DFOT) and deep $g'r'i'$ exposures with the CAHA telescope. At late times, we targeted the field with the Large Monolithic Imager (LMI) on the 4.3-m Lowell Discovery Telescope (LDT) using the u, g, r, i, and z filters to refine the photometry of its host galaxy.

The data were pre-processed using standard CCD reduction techniques including bias subtraction, flat-fielding, fringe correction and cosmic-ray rejection. Aperture photometry on the GRB counterpart was performed using Source Extractor[88] and selecting circular apertures with radius 1.5 times larger than the image full width half maximum. Forced photometry was performed on images acquired at later times (>T_0 + 2 d). Host galaxy magnitudes were derived using Kron-like elliptical apertures (MAG AUTO). The photometric zeropoints were calibrated using the same nearby point sources in the Sloan Digital Sky Survey (SDSS[89]) and transformed using empirical equations[90].

We imaged the field with the *Hubble Space Telescope* (*HST*) using the WFC3/UVIS and IR cameras with the *F814W* and *F160W* filters, respectively. Observations were carried out between April 2 and April 21, 2022 (~T_0 + 4 months). The data were reduced using standard procedures within the DrizzlePac software[91] to align, drizzle, and combine exposures. We used Source Extractor to detect sources and perform aperture photometry. For each filter, we used the zeropoints stored in the keyword PHOTFLAM.

Gemini Spectroscopy - We utilized the Gemini Multi-Object Spectrograph (GMOS) mounted on the 8.1-m Gemini South telescope to obtain a series of 4×600 s spectra, using the R400 grating and a 1" slit. We chose a slit position angle (PA) of 64 deg such that the slit covers both the host galaxy's center and the GRB's optical position. The data were reduced and analyzed using standard procedures in Gemini IRAF. In order to correct for slit losses, the flux-calibrated spectra were matched to the photometry of the host galaxy. Line fluxes were derived by fitting each line with a Gaussian function and estimating the continuum from nearby spectral regions.

White dwarf merger model

One of the possible progenitors adopted to explain SN-less long GRBs is the merger of a white dwarf (WD) with either a NS or a stellar mass BH[92,93]. These are old stellar systems, and their mergers produce accretion disks with longer accretion timescales than those from compact binary mergers[94]. Therefore, they can potentially explain the lack of SN, the long gamma-ray duration, and the environment of hybrid GRBs. The merger ejecta contains a moderate amount (<0.1 M_{sun}) of radioactive ^{56}Ni powering fast-evolving (weeks to month long) optical transients[95] with luminosities in the range 10^{40} - 10^{43} erg s^{-1}. However, past light curve calculations[96] of these electromagnetic counterparts resemble faint type Iax SNe, and do not match the colors and timescales of the excess emission in GRB211211A.

Rate of events

In 17 years of *Swift* operations at least 2 hybrid GRBs were identified, GRB211211A and GRB060614. This allows us to place a lower limit to the rate of hybrid GRBs:

$$R > 0.7 \frac{\Omega}{4\pi}\frac{1}{V_z}\frac{1}{\varepsilon T}\frac{1}{\eta} \approx 0.04\, Gpc^{-3}\, yr^{-1} \qquad (1)$$

where Ω~ 2.2 sr is the *Swift* field of view for partial coding >10%, T ~ 17 yr the mission lifetime, ε ~78% its duty cycle[11], η ~ 17/20 the efficiency of SN searches, and V_z ~ 7.3 Gpc^3 the

comoving volume within z~0.3. The factor 0.7 is the lower limit (68% confidence level, c.l.) on the number count derived from Poissonian statistics[97].

In a similar way we derive an upper limit to the rate by assuming that all the SN-less long GRBs within z~0.3 belong to the class of nearby hybrid bursts. We consider events with $E_{\gamma,iso} > 10^{49}$ erg in order to minimize selection effects due to the trigger efficiency. This only excludes one burst (GRB111005A). The 68% c.l. upper limit on the number count is ~12, from which we derive $R < 0.8$ Gpc^{-3} yr^{-1} using Supplementary Equation (1).

For comparison, the observed rate of short GRBs[40] ranges between 2.2 Gpc^{-3} yr^{-1} and 6.4 Gpc^{-3} yr^{-1} (68% c.l.). This value was derived for luminosities $L_{iso} > 5 \times 10^{49}$ erg s^{-1}, and therefore does not include the contribution of under-luminous off-axis bursts. The observed ratio of hybrid to short GRBs is simply given by the ratio of the two distributions (0.8%-26% at the 68% c.l.). However, the local population of GW counterparts is likely dominated by faint events seen off-axis[98], whose rate of detection depends on the distribution of jet opening angles and their angular profiles. We parameterize these properties using the beaming factor f_b, and caution that its value may differ between the population of bursts (hybrid and short), thus affecting their relative ratio in the nearby Universe.

Supplementary Table

Supplementary Table 1 – UV, optical and nIR observations of GRB211211A.

Upper limits (u.l.) are 3σ. Values are corrected for Galactic extinction in the direction of the burst, $A_V = 0.047$ mag[99].

T - T_0 (d)	Exposure (s)	Telescope	Filter	Magnitude AB	Error (68% c.l.)
0.0009	9	UVOT	*v*	17.3	u.l.
0.0012	36	UVOT	*White*	20.33	0.25
0.0020	96	UVOT	*White*	21.2	u.l.
0.042	195	UVOT	*u*	19.75	0.13
0.044	193	UVOT	*b*	19.62	0.2
0.046	185	UVOT	*White*	19.6	0.06
0.049	196	UVOT	*w2*	19.59	0.12
0.051	195	UVOT	*v*	19.27	0.28
0.053	196	UVOT	*m2*	19.58	0.18
0.056	196	UVOT	*w1*	19.44	0.12
0.058	78	UVOT	*u*	19.38	0.16
0.19	877	UVOT	*u*	19.75	0.07
0.20	570	UVOT	*b*	19.79	0.17
0.24	3300	MITSUME	*g*	19.85	0.15
0.24	3300	MITSUME	R_c	20.3	0.19
0.24	3300	MITSUME	I_c	20.2	u.l.
0.25	467	UVOT	*m2*	20.5	0.16
0.29	3300	MITSUME	*g*	20.19	0.16
0.29	3300	MITSUME	R_c	19.99	0.18
0.29	3300	MITSUME	I_c	19.98	0.19
0.37	200	DOT	*R*	20.17	0.06
0.38	300	DOT	*I*	20.26	0.08
0.38	300	DOT	*R*	20.03	0.05
0.38	300	DOT	*V*	20.18	0.07
0.39	300	DOT	*B*	20.51	0.07
0.39	360	DOT	*U*	20.78	0.07
0.40	300	DOT	*I*	20.17	0.08
0.40	300	DOT	*R*	20.14	0.04
0.40	300	DOT	*V*	20.26	0.04
0.41	300	DOT	*B*	20.45	0.06

0.41	360	DOT	*U*	20.75	0.07
0.42	200	DOT	*I*	20.20	0.09
0.42	200	DOT	*R*	20.15	0.05
0.42	200	DOT	*V*	20.28	0.04
0.42	200	DOT	*B*	20.49	0.05
0.43	360	DOT	*U*	20.76	0.07
0.43	200	DOT	*I*	20.08	0.08
0.43	200	DOT	*R*	20.24	0.04
0.44	360	DOT	*U*	20.81	0.07
0.45	200	DOT	*I*	20.17	0.09
0.45	200	DOT	*R*	20.29	0.05
0.45	200	DOT	*V*	20.35	0.05
0.46	200	DOT	*B*	20.53	0.05
0.46	360	DOT	*U*	20.97	0.07
0.46	200	DOT	*I*	20.34	0.09
0.47	200	DOT	*R*	20.41	0.09
0.47	200	DOT	*V*	20.27	0.07
0.47	200	DOT	*B*	20.68	0.15
0.62	900	CAHA	*i*	20.71	0.09
0.63	900	CAHA	*r'*	20.73	0.09
0.64	720	CAHA	*g'*	21.16	0.08
0.75	1771	UVOT	*w1*	21.96	0.19
0.81	1771	UVOT	*w2*	22.32	0.20
0.83	370	UVOT	*v*	19.8	u.l.
0.93	1247	UVOT	*u*	22.15	u.l.
0.98	1949	UVOT	*b*	22.3	0.4
1.22	1422	UVOT	*w1*	22.3	u.l.
1.26	813	UVOT	*m2*	22.2	u.l.
1.26	6480	MITSUME	*g*	20.5	u.l.
1.26	6480	MITSUME	R_c	20.8	u.l.
1.26	6480	MITSUME	I_c	20.4	u.l.
1.41	900	DOT	*R*	22.54	0.09
1.42	900	DOT	*I*	22.1	0.15
1.43	1200	DOT	*V*	23.09	0.19
2.7	2550	CAHA	*i'*	24.51	0.28
2.9	4775	UVOT	*m2*	23.5	u.l.

3.4	3600	DOT	*R*	24.4	u.l.
4.0	900	Gemini[100]	*K*	22.4	0.1
4.4	3900	DOT	*R*	25.2	u.l
6.9	3780	MMT[101]	*K*	23.9	0.3
20.6	4000	CAHA	*i'*	23.2	u.l.
20.7	3200	CAHA	*r'*	24.4	u.l.
20.7	800	CAHA	*g'*	23.6	u.l.
22.5	7200	DFOT	*R*	24	u.l.
		Host Galaxy			
142	1495	UVOT	*w2*	21.64	0.14
2.9	4775	UVOT	*m2*	21.52	0.15
140	2420	UVOT	*w1*	21.50	0.18
0.2	877	UVOT	*u*	21.08	0.18
0.5	200	DOT	*B*	20.14	0.10
1.4	200	DOT	*V*	19.60	0.10
3.4	200	DOT	*R*	19.46	0.05
22.5	7200	DFOT	*R*	19.50	0.10
131	1400	LDT	*u*	20.95	0.04
172	300	LDT	*g*	19.93	0.01
172	300	LDT	*r*	19.50	0.01
131	600	LDT	*i*	19.20	0.01
131	600	LDT	*z*	19.07	0.02
111	2160	*HST*	*F814W*	19.24	0.01
114	4823	*HST*	*F160W*	18.93	0.01
Archival	–	WISE[102]	*W1*	19.88	0.05
Archival	–	WISE	*W2*	20.68	0.19

Additional References

77. Evans, P. A. et al., Methods and results of an automatic analysis of a complete sample of Swift-XRT observations of GRBs. *Mon. Not. R. Astron. Soc.* **397**, 1177-1201 (2009)
78. Breeveld, A. A. et al., Further calibration of the Swift ultraviolet/optical telescope. *Mon. Not. R. Astron. Soc.* **406**, 1687-1700 (2010)
79. Kotani, T. et al. MITSuME---Multicolor Imaging Telescopes for Survey and Monstrous Explosions. *Nuovo Cimento C Geophysics Space Physics C.* **28**, 755 (2005)
80. Yatsu, Y. et al. Development of MITSuME—Multicolor imaging telescopes for survey and monstrous explosions. *Physica E Low-Dimensional Systems and Nanostructures.* **40**, 434-437 (2007)
81. Shimokawabe, T. et al. MITSuME: multicolor optical/NIR telescopes for GRB afterglows. *AIP Conf. Ser.* **1000**, 543-546 (2008)
82. Niwano, M. et al. A GPU-accelerated image reduction pipeline. *Publications of the Astronomical Society of Japan.* **73**, 14-24 (2021)
83. Becker, A. HOTPANTS: High Order Transform of PSF ANd Template Subtraction. Astrophysics Source Code Library. ascl:1504.004 (2015)
84. Kumar, B. et al. 3.6-m Devasthal Optical Telescope Project: Completion and first results. *Bulletin de la Societe Royale des Sciences de Liege.* **87**, 29-41 (2018)
85. Pandey, S. B. et al. First-light instrument for the 3.6-m Devasthal Optical Telescope: 4Kx4K CCD Imager. *Bulletin de la Societe Royale des Sciences de Liege.* **87**, 42-57 (2018)
86. Kumar, A. et al. Photometric calibrations and characterization of the 4Kx4K CCD Imager, the first-light axial port instrument for the 3.6m DOT. Preprint at https://arxiv.org/abs/2111.13018 (2021)
87. Gupta, R. et al., GRB 211211A: Observations with the 3.6m Devasthal Optical Telescope. *GCN Circ.* **31299** (2021)
88. Bertin, E. & Arnouts, S. SExtractor: software for source extraction. *Astron. Astrophys. Suppl. Ser.* **117**, 393–404 (1996)
89. Ahumada, R. et al. The 16th Data Release of the Sloan Digital Sky Surveys: First Release from the APOGEE-2 Southern Survey and Full Release of eBOSS Spectra. *Astrophys. J. Sup. Ser.* **249**, 3 (2020)
90. Jordi, K., Grebel, E. K. & Ammon, K. Empirical color transformations between SDSS photometry and other photometric systems. *Astron. Astrophys.* **460**, 339-347 (2006)
91. Gonzaga, S., Hack, W., Fruchter, A. & Mack, J. *The DrizzlePac Handbook* (STScI, Baltimore, 2012)
92. Dong, Y.-Z., Gu, W.-M., Liu, T. & Wang, J. et al. A black hole-white dwarf compact binary model for long gamma-ray bursts without supernova association. *Mon. Not. R. Astron. Soc.* **475**, L101-L105 (2018)
93. Caito, L. et al. GRB060614: a ``fake'' short GRB from a merging binary system. *Astron. Astrophys.* **498**, 501-507 (2009)
94. Fryer, C. L., Woosley, S. E. & Hartmann, D. H. Formation Rates of Black Hole Accretion Disk Gamma-Ray Bursts. *Astrophys. J.* **526**, 152-177 (1999)
95. Gillanders, J. H., Sim, S. A. & Smartt, S. J. AT2018kzr: the merger of an oxygen-neon white dwarf and a neutron star or black hole. Mon. Not. R. Astron. Soc. **497**, 246-262 (2020)
96. Bobrick, A. et al. Transients from ONe white dwarf - neutron star/black hole mergers. *Mon. Not. R. Astron. Soc.* **510**, 3758-3777 (2022)
97. Gehrels, N. Confidence Limits for Small Numbers of Events in Astrophysical Data. *Astrophys. J.* **303**, 336 (1986)

98. Dichiara, S. et al. Short gamma-ray bursts within 200 Mpc. *Mon. Not. R. Astron. Soc.* **492**, 5011-5022 (2020)
99. Schlafly, E. F. & Finkbeiner, D. P. Measuring Reddening with Sloan Digital Sky Survey Stellar Spectra and Recalibrating SFD. *Astrophys. J.* **737**, 103 (2011)
100. Levan, A., et al. GRB 211211A - Gemini K-band detection. *GCN Circ.* **31235** (2021)
101. Rastinejad, J. et al. MMT/MMIRS Observations Indicate Fading of K-band Source. *GCN Circ.* **31264** (2021)
102. Marocco F. et al. The CatWISE2020 Catalog. *Astrophys. J. Sup. Ser.*, **253**, 8 (2021)
